# SDU – software for high-throughput automated data collection at the Swiss Light Source

**DOI:** 10.1107/S1600577523002631

**Published:** 2023-04-12

**Authors:** Kate Mary Louise Smith, Ezequiel Panepucci, Jakub Wojciech Kaminski, Sylvain Aumonier, Chia-Yiang Huang, Deniz Eris, Dominik Buntschu, Nathalie Meier, Wayne Glettig, Katherine Evelyn McAuley, Meitian Wang, May Elizabeth Sharpe, Justyna Aleksandra Wojdyla

**Affiliations:** a Swiss Light Source, Paul Scherrer Institute, 5232 Villigen PSI, Switzerland; RIKEN SPring-8 Center, Japan

**Keywords:** beamline automation, loop centering, protein crystallography, data acquisition software, high throughput

## Abstract

Recent advances in automation have fostered the development of unattended data collection services at a handful of synchrotron facilities worldwide. At the Swiss Light Source, the installation of new high-throughput sample changers at all three macromolecular crystallography beamlines and the commissioning of the Fast Fragment and Compound Screening pipeline created a unique opportunity to automate data acquisition. Here, the DA+ microservice software stack upgrades, implementation of an automatic loop-centering service and deployment of the Smart Digital User (SDU) software for unattended data collection are reported. The SDU software is the decision-making software responsible for communications between services, sample and device safety, sample centering, sample alignment with grid based X-ray diffraction and, finally, data collection.

## Abstract 2

Fully automated data collection is now available at the Swiss Light Source macromolecular crystallography beamlines using the Smart Digital User (SDU) software.

## 1. Introduction

Recent advances in the macromolecular crystallography (MX) beamline hardware instrumentation, such as high-capacity and high-throughput sample changers (Martiel *et al.*, 2020 ▸; O’Hea *et al.*, 2018 ▸; Papp *et al.*, 2017 ▸; Russi *et al.*, 2016 ▸) and the latest pixel array detectors (Dinapoli *et al.*, 2011 ▸; Casanas *et al.*, 2016 ▸; Johnson *et al.*, 2014 ▸), have led to an increased sample throughput with more than 20 samples measured within an hour of beam time becoming a standard for both industry and academic users. In parallel with hardware, substantial effort has been put into the development of sophisticated data acquisition software and graphical user interfaces (GUIs), such as *Blu-Ice* (McPhillips *et al.*, 2002 ▸), *BSS* (Ueno *et al.*, 2005 ▸), *CBASS* (Skinner *et al.*, 2006 ▸), *STARS* (Yamada *et al.*, 2008 ▸), *mxCUBE* (Gabadinho *et al.*, 2010 ▸), *JBluIce-EPICS* (Stepanov *et al.*, 2011 ▸), *GDA* (Winter & McAuley, 2011 ▸), *MxDC* (Fodje *et al.*, 2012 ▸) and *Bluesky* (Allan *et al.*, 2019 ▸). These ongoing hardware and software developments have resulted in a streamlined experience for users performing different types of MX data collection.

In the past few years MX experiment methodology has experienced clear branching. On the one hand, beamline staff focused on development and support of more sophisticated types of experiments, such as serial crystallography, pump–probe, temperature gradient, and new sample delivery modes, namely jet, fixed target and levitation (Oghbaey *et al.*, 2016 ▸; Martiel *et al.*, 2019 ▸; Huang *et al.*, 2022 ▸; Kepa *et al.*, 2022 ▸; Pearson & Mehrabi, 2020 ▸; Keedy *et al.*, 2015 ▸). On the other, standardization and high throughput of standard rotational and the more mature fixed target experiments have become the holy grail of many MX beamlines. Moreover, following in the long-established footsteps of the pharmaceutical industry, many synchrotrons have developed in-house X-ray crystallography fragment screening pipelines which are available to academic users as well as to pharmaceutical companies (Douangamath *et al.*, 2021 ▸; Kaminski *et al.*, 2022 ▸; Lima *et al.*, 2020 ▸). Large quantities of samples created as part of high-throughput screening and fragment-based lead discovery processes are a perfect target for unattended data collection routines. The success of automation relies on one major prerequisite – robust and reliable beamline hardware. Of particular importance are beam position, robotic sample mounting system, detector and sample environment lighting. The latter is essential to enable successful automatic loop centering based on image processing (Lavault *et al.*, 2006 ▸; Jain & Stojanoff, 2007 ▸; Ito *et al.*, 2019 ▸; Gaponov *et al.*, 2016 ▸; Hirata *et al.*, 2019 ▸). The first fully automated data collection routines, which were aimed mostly at pharmaceutical industry clients, were implemented in the USA at the Advanced Photon Source LRL-CAT beamline (Wasserman *et al.*, 2012 ▸) and the Stanford Synchrotron Radiation Lightsource (Tsai *et al.*, 2013 ▸). Recently there was an ambitious initiative at the European Synchrotron Radiation Facility (ESRF) MASSIF-1 beamline (Svensson *et al.*, 2015 ▸, 2018 ▸), which incorporates full sample characterization for optimal data collection strategy. This has been followed by the continued development and expansion of *GDA* to include unattended data collection (UDC) at Diamond Light Source (Vollmar & Evans, 2021 ▸; Winter & McAuley, 2011 ▸). The application of UDC software has also been extended to serial crystallography with the development of *ZOO* at SPring-8 (Hirata *et al.*, 2019 ▸).

The Swiss Light Source (SLS) Macromolecular Crystallography Group operates three beamlines (X06SA, X06DA and X10SA). SLS MX beamlines are served by an in-house-developed distributed DA+ software stack, which supports standard rotational single crystal and serial crystal data acquisition and analysis (Wojdyla *et al.*, 2018 ▸; Basu *et al.*, 2019 ▸). Recent SLS MX hardware developments include the TELL robot with increased dewar capacity and sample exchange speed (Martiel *et al.*, 2020 ▸), new TELL gripper design with pin detection, implementation of the fast fragment- and compound-screening pipeline (FFCS) (Kaminski *et al.*, 2022 ▸), upgrade of the sample environment top camera and back light, and the installation of SmarGon MCS2 with in-house smargopolo controls software and calibration routine (Glettig *et al.*, 2022 ▸).

In this paper we describe how we have leveraged these latest developments and extended the DA+ software microservice architecture with TELL sample spreadsheet data model validation, deployed automated loop centering, provided users with expanded automatic data processing routines (ADP native-sad merging), migrated our database to the cloud, and implemented a sophisticated communications and decision making software, Smart Digital User (SDU), for fully automated data collection.

## 2. Automatic loop centering

The automatic loop centering (ALC) microservice implemented at all three MX beamlines at SLS (X06DA, X06SA, X10SA) is written in Python 3.9 (Van Rossum & Drake, 2009 ▸) with a RESTful API server/client architecture. The ALC microservice uses the open computer vision (openCV; https://opencv.org/) package for identification of loop tips, NumPy (https://numpy.org/) for pixel conversion and motor movement calculations, and pyepics (https://pyepics.github.io/pyepics/) for motion control. The ALC Flask server (https://palletsprojects.com/p/flask/) operates as a state machine and utilizes redis (https://redis.io/) to ensure thread safety, *i.e.* execution of a single centering worker at a time to ensure correct and safe movements of motors. At the X06SA and the X10SA beamlines, ALC consists of two core steps: (i) pre­location and (ii) microscope centering. The X06DA beamline setup varies slightly as the sample microscope field of view is sufficiently wide to enable loop centering without the pre­location step. For the ALC software to be successful there are important hardware considerations and setup requirements. It is important to have stable hardware, good lighting to fully illuminate the sample, correct pixel-to-millimetre conversion for each camera, and beamline-specific openCV parameters.

### 2.1. ALC prelocation

**Figure 1:**
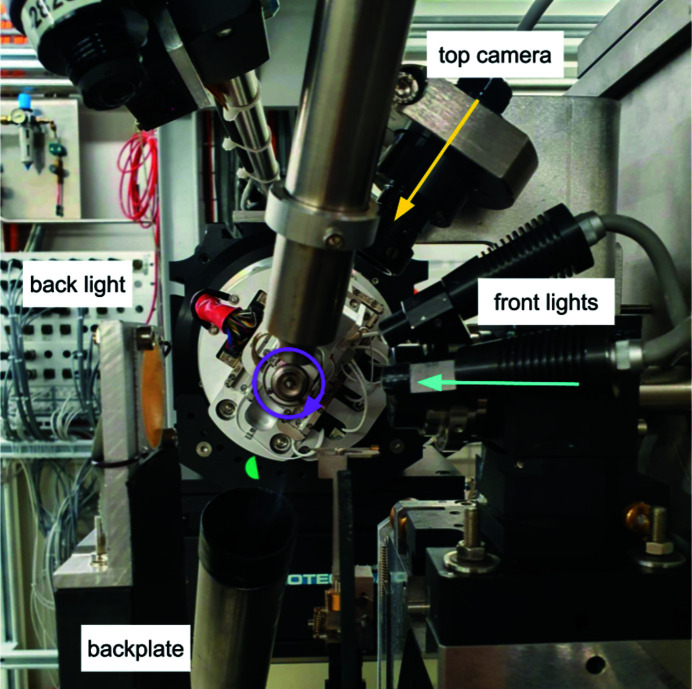
ALC hardware setup in the sample environment. Photograph of the X06SA beamline stable mounted top camera, front lights, back light and backplate. The X-ray beam direction is shown in cyan, the ω rotation axis is shown in magenta, and the direction of the top camera for preliminary optical alignment is shown in yellow (perpendicular to the ω rotation axis). The top camera mount CAD-drawings are available in Fig. S1 of the supporting information.

Even at the lowest magnification setting, the microscope sample cameras at X06SA and X10SA do not provide a field of view wide enough to visualize pins of different lengths and at diverse angles. Therefore, to ensure a successful loop prelocation, a secondary camera was mounted above the sample environment, perpendicular to the omega rotation axis, with a focus on the area encompassing the tip of the mounted sample (Fig. 1 ▸). The realization of sample prelocation relies on adequate sample lighting and a uniform background, provided by the dedicated front light (mounted on top of the sample microscope) and a black plate mounted onto the back light. Another important consideration is the correct mapping of the beam position onto the top camera. During routine beamline startup prior to user operation, beam position is marked onto the sample microscope image and subsequently mapped onto the top camera image with the *calibrateTopCam* worker. This calibration procedure is fully automated, takes 4 s to complete, and is incorporated into the DA+ GUI expert panel. Since the installation of the new stable camera mount one year ago we have tracked the variance in beam position. Although we have observed a variation of only 7 pixels in *X* position and 2 pixels in *Y* position (within two standard deviations), we have integrated the procedure into the routine beamline setup to ensure as accurate a position as possible prior to the commencement of experiments, as it is a very quick and straightforward procedure.

The ALC *prelocate* program (i) identifies the tip of the loop; (ii) moves the tip to the beam position; (iii) rotates the sample 90°; and (iv) repeats steps (i) and (ii), thus aligning the sample and rotation axis to the beam position. This step is critical for the microscope centering stage as it ensures the sample is in focus and within the microscope camera field of view at high magnification (small ROI and shallow depth of focus).

### 2.2. ALC microscope centering

**Figure 2:**
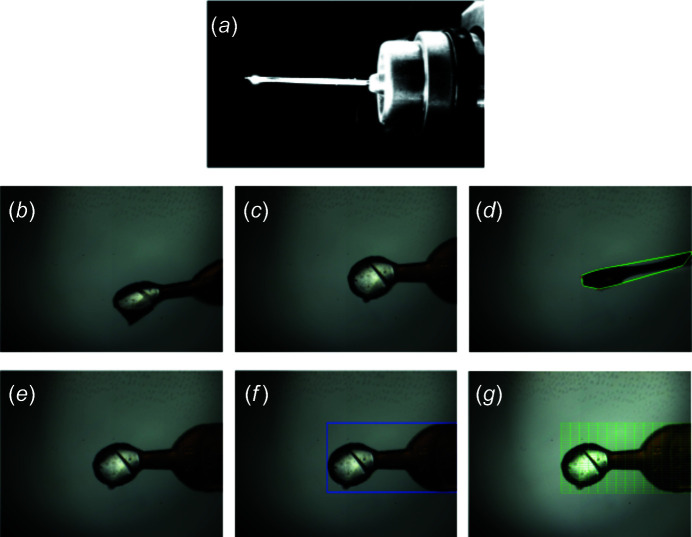
ALC steps. (*a*) Image from the top camera after the prelocation procedure, which aligns the sample to the rotation axis and beam position to ensure (*b*) the sample camera view is in-focus. *centerToFlat* procedure, which (*c*) firstly centers the sample to the beam position and rotates the sample every 40° to find the flat face of the loop; (*d*) rotates the sample 90° to find the loop thickness; (*e*) centers the sample once more to the beam position and returns the loop to the flat face angle and moves it to the center of ROI; and (*f*) determines the bounding box and returns the pixel coordinates of the bounding box corners and motor coordinates of the loop tip. (*g*) ALC *centerToFlat* results are used to calculate the grid scan positions in SDU.

Two core classes were implemented as part of the ALC centering with the sample camera – *centerOnTip* and the more extensive *centerToFlat* procedure (Fig. 2 ▸). The default setting for manual data collection is *centerOnTip* as it is the fastest method to bring the loop to beam position. The ALC movements occur asynchronously with the state change of the beamline from robot sample exchange to user sample alignment mode. Consequently, the *centerOnTip* procedure is completed before a user is able to begin manual alignment of their sample. The full optical centering with *centerToFlat* is utilized primarily for automation, although some users also favor this option for their interactive work. For users preferring to center their samples manually and for special applications (*e.g.* chips for serial crystallography), we provide options to disable the initial prelocation step and subsequent centering (*noCentering*) in the DA+ GUI.

The *centerOnTip* procedure first locates the loop tip and aligns it to the beam marker. It then performs a 90° rotation and aligns the tip once again to the beam marker, which takes ∼4 s to perform. The *centerToFlat* procedure first runs *centerOnTip*, and after the tip has been centered the sample is rotated by a total of 180° with images taken at 40° apart. This allows the calculation of a sinusoidal curve to determine the maximum area of the sample with *findFlatFace*. The sample is subsequently rotated 90°, and the height (thickness) of the sample is determined with *findLoopThickness*. The height was traditionally used for the calculation of the vertical grid scan size. However, we realized that in the case of heavily icy pin bases, despite the usage of compressed air to dry them, it is quite common for slight movements of the sample to occur between the loop centering and X-ray diffraction centering step. Therefore, it is more practical to vertically scan the entire ROI rather than to use the loop thickness to restrict the vertical grid scan size, which can occasionally result in missing part of the sample. The *centerOnTip* procedure is again performed to ensure the sample tip and rotation axis are aligned to the beam position. Finally, *findLoopBoundingBox* rotates the sample 90° to the flat face position to determine the bounding rectangle covering the whole sample that is used for the 2D raster scan. The entire *centerToFlat* procedure takes approximately 11 s to perform.

### 2.3. ALC results

The *centerToFlat* output (motor positions and bounding box pixel coordinates) is retrieved by SDU. This information is used in conjunction with the ALC *beamlineMotors* class to generate motor positions for grid scanning, which can be visualized in the DA+ GUI for debugging purposes without X-rays. *saveRasterGrid* receives spot finding results from SDU to overlay a heat map on the current sample image from the microscope. ALC also contains additional workers such as *saveCurrentImage*, which is used by the DA+ GUI and SDU to request an image of the sample from the microscope camera prior to data acquisition.

## 3. SDU

In combination with existing DA+ software infrastructure (ALC, TELL GUI, DA+ server, mxdb *etc*.), the in-house developed SDU software enables fully automated data collection at all three SLS MX beamlines. The SDU iterates over rows of the user-provided spreadsheet, where every row corresponds to a single sample. Each sample is: mounted onto a goniometer with the TELL sample changer; optically centered with the ALC; X-ray diffraction centered with a 2D grid scan, and subsequent line-scan 90° away from the 2D grid scan to identify the ‘best’ spot for data collection; then the X-ray diffraction data set is collected upon using user-provided or default parameters; and, finally, data are automatically processed according to user-provided (or default; see Table S3 of the supporting information) instructions.

### 3.1. SDU structure and communication

**Figure 3:**
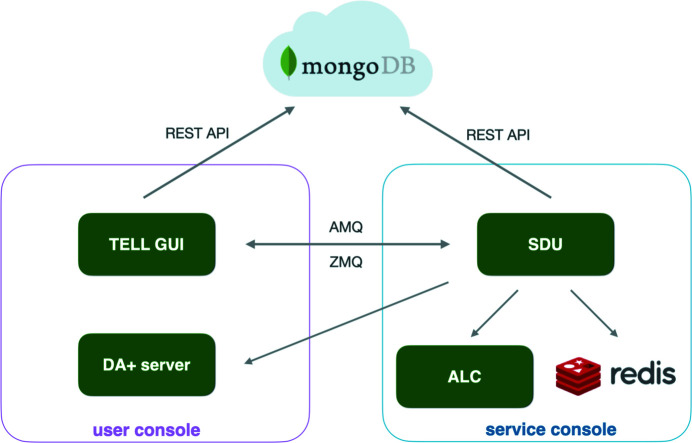
SDU incorporation into the DA+ software stack. Software instances which are represented as green boxes are located on the beamline-specific user console (outlined with the magenta box) and service console (outlined with the blue box). The MongoDB mxdb database is located on the cloud Microsoft Azure cluster. Communication between software microservices is indicated with gray arrows, with the SDU–TELL GUI–mxdb triangle modes specified.

SDU is a multi-threaded Python 3.6 application embedded within the existing DA+ software stack. It utilizes a redis key-value database to ensure asynchronous and error-free interaction with other software instances. SDU communicates with other components of the DA+ software stack *via* dedicated REST APIs, an ActiveMQ Classic message broker (https://activemq.apache.org/) and the ZeroMQ messaging library (https://zeromq.org/). The two main SDU interaction partners are the TELL GUI and the MongoDB mxdb database, which form the ‘data collection – live progress tracking – information storage’ triangle (Fig. 3 ▸). The triangle is designed to ensure recovery of the DA+ software stack in case one of the components fails. The full state of the experiment, including measured and mounted samples, is stored in both the mxdb cloud database and as a cache in redis. In the event of a server or application crash, following recovery of the appropriate services the TELL GUI is able to continue measurements from the point of failure.

The main SDU execution class, *SduExecutor*, performs automation on the given sample, *i.e.* row from the currently active spreadsheet. *SduExecutor* first identifies the SDU data collection method and steps of the execution stack, which depend on the type of data collection. Currently, SDU accepts standard, multi-orientation and simplified serial crystallography (under development) methods which can be specified in the sample spreadsheet on a per sample basis. The following SDU steps are implemented (programmed as Python classes):

(i) *safety_check* – ensures that the beamline hardware and software are in a correct faultless state before SDU progresses further.

(ii) *mount* – sends a sample mount request to the TELL robot and awaits a return message with the mount status, which is evaluated accordingly.

(iii) *optical_center* – retrieves results of the ALC which was initiated by the TELL GUI after a successful sample mount, and passes them on to the *diff_center* step.

(iv) *diff_center* – performs 2D and linear grid scans (omitted in the case of the simplified serial crystallography method). This step assembles data collection messages based on the ALC results and sends data acquisition requests to the DA+ server. It also collects and analyzes the grid scan diffraction image processing results to identify the best spot for data collection (analysis options shown in Table S2 of the supporting information).

(v) *daq* – assembles the data acquisition message which is sent to the DA+ server. Message content is based on a selected method and data collection parameters (either provided by the user in the sample spreadsheet or default values, shown in Table S1 of the supporting information). This step also monitors data writing to the local file storage.

*SduExecutor* was engineered to allow some degree of freedom. For example, by default the standard method includes all the aforementioned steps, but it can also consist of a *safety_check* and mount execution stack, which are used for automated testing of the TELL robot mounting of multiple samples. Extending the *safety_check*, mount stack with an *optical_center* step, allows for automated ALC testing, which is performed on a set of pucks with a selection of varied loop types.

After the execution stack is established, *SduExecutor* first checks the redis status to ensure SDU is ready to proceed (and not paused by a user or in a failure status due to a beamline issue); it then updates redis with the current row and executes the SDU steps. Each step updates the SDU progress status stored in the redis and the mxdb databases and sends the ActiveMQ messages to dedicated TELL GUI topics. *SduExecutor* is able to catch errors and failed tasks in which case automation is immediately stopped. In the case of serious hardware issues, an email, SMS and an optional WhatsApp notification is sent to authorized staff who can resume SDU after beamline recovery.

### 3.2. User interface and experiment tracking

**Figure 4:**
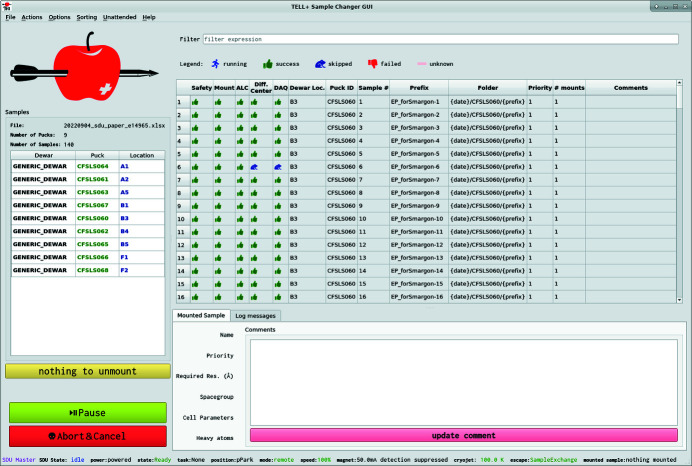
TELL GUI Unattended tab view with results of the SDU run.

The TELL GUI, which enables sample mounting with the TELL automatic sample changer (Martiel *et al.*, 2020 ▸), was extended for on-the-fly display of the SDU automation progress. For that purpose a dedicated ‘Unattended’ tab was developed and incorporated into the TELL GUI. Similarly to the manual operation view, the Unattended tab includes a list of samples and additional columns which correspond to the SDU execution stack steps (labeled ‘Safety’, ‘Mount’, ‘ALC’, ‘Diff Center’ and ‘DAQ’), as well as two buttons (‘Start’ and ‘Abort & Cancel’) which allow control of the SDU run. The status of the TELL GUI is shown at the bottom left corner as ‘SDU master’ when it is in unattended mode (Fig. 4 ▸). While the automated procedure progresses through the SDU steps, the status is updated in the TELL GUI with the appropriate icon. The SDU steps can be in progress (blue running man), successfully completed (green thumbs up), skipped (blue frog), failed (red thumb down) or unknown (beige minus; default TELL GUI view before the SDU run is started).

During the SDU diffraction centering step, grid scan diffraction images are processed on-the-fly as described previously (Wojdyla *et al.*, 2016 ▸). In the case of manual data collection, grid scan processing results are received and displayed by the DA+ GUI for manual user interaction, while during automation SDU receives these processing results. SDU aggregates results from all grid boxes into a single data object, which is subsequently analyzed to identify the best spot for data collection. During analysis of 2D and vertical line grid boxes, SDU applies thresholding. Apart from default thresholding parameterization, which works for most samples, additional options were implemented for strong (well diffracting crystals), weak (weakly diffracting crystals such as membrane proteins) and always-collect (if no grid box) scenarios. If the number of spots for all grid scan boxes (in the case of both 2D and vertical line grids) is below the threshold, data collection for this sample is skipped (indicated with the frog icon in the TELL GUI and SDU report), and SDU proceeds to mount the next sample.

**Figure 5:**
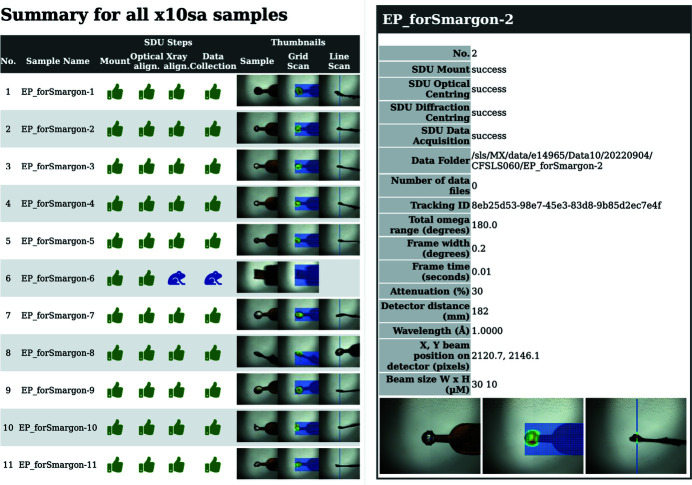
SDU final report. Summary table for all samples and single sample report page.

At the end of SDU automated data collection, TELL GUI automatically generates a report (in .html and .pdf format), which includes a summary table and a more detailed single-page information sheet for each sample. The summary table includes the status of each step of the SDU execution stack indicated with the appropriate icon (following the same format as implemented in the TELL GUI) and three image thumbnails – ALC sample image and overlays of 2D and line-scan grid scan results represented as a heat map (Fig. 5 ▸). The single-page information sheet includes selected data collection parameters such as data folder, exposure time and oscillation, as well as larger versions of thumbnails presented in the summary table (Fig. 5 ▸). The SDU report is complemented by the web-generated ADP report (.pdf format), which contains a full data processing summary. The users are able to utilize the unattended TELL GUI to go back and check crystals manually if needed. To obtain experimental data all users are able to scp or rsync their files from our remote access cluster, or use GridFTP with GlobusOnline. Academic users are also able to manually submit their data for long-term tape storage with the SciCat data catalog (https://discovery.psi.ch).

### 3.3. Recent DA+ software updates

Since the first publication of the DA+ distributed software stack (Wojdyla *et al.*, 2018 ▸; Basu *et al.*, 2019 ▸), many upgrades have taken place in order to support the development of SDU. The Python beamline library mxlibs (Python2.7), which abstracts standard and non-standard epics motors, was recently replaced by mxlibs3 (Python3.9), which utilizes pyepics. The Python escape workflow engine and data acquisition server were also updated during this migration. As more automation was added to the distributed software stack, consistency of information transfer between these distinct software packages became a paramount concern. To address this, pydantic data models (https://github.com/samuelcolvin/pydantic) were added to mxlibs3 for validation of the TELL sample spreadsheet, which contains information for sample mounting (TELL), data acquisition (DA+ GUI, SDU) and data processing (ADP, ADM, dimmer). The spreadsheet validation occurs both prior to the experiment within a Vue2 web application and at the beginning of the experiment within the TELL GUI. Recently, the mxdb in-house MongoDB docker instance was upgraded to a MongoDB Atlas fully managed cloud database service. The new mxdb runs as a three-node replica set, providing redundancy and high availability on a Microsoft Azure cluster. All information is encrypted both at rest and in transit, with the mxdbserver securely transferring information between the cloud database and our beamline applications (TELL GUI, ADP, DA+ server *etc*.).

## 4. SDU automated advanced collection protocol

In 2022, the SmarGon MCS2 was installed at both X06SA and X10SA beamlines with the in-house-developed controls software smargopolo and calibration procedures (Glettig *et al.*, 2022 ▸). The SmarGon MCS2 goniometer enables the collection of multi-orientation datasets within the angular ranges 0–40° (χ) and ±360° (φ) without the requirement of re-centering the sample as the achieved sphere of confusion for χ is 4 µm and for φ is ∼1.5 µm (Glettig *et al.*, 2022 ▸). It has been shown that multi-angle dataset collections increase data quality for experimental phasing (Weinert *et al.*, 2015 ▸) and improve electron density maps for ligand studies (Bricogne *et al.*, 2018 ▸). Although performing a fragment screening campaign and data processing analysis is not within the scope of this paper, we would like to present the recently implemented SDU and ADP extension, which allows the utilization of SmarGon MCS2 goniometer features. Multi-orientation SDU dataset collections can be defined in the spreadsheet as a list of χ,φ pairs. Following SDU data collection, ADP software automatically merges the single-crystal multi-orientation datasets together using the first dataset as a reference. To showcase the new features, we have performed a small-scale multi-angle FFCS experiment using an endothia­pepsin crystallization system to provide statistics on the data acquisition speeds of collections using single-axis and multi-orientation with two χ angles. For multi-orientation dataset collection at χ = 0° and χ = 20°, the measurement speed for samples at X10SA was approximately 19 samples per hour, as compared with 20 samples per hour for standard collection. Overall, on the data acquisition side, there are slight speed trade-offs between collecting multi-orientation versus single-orientation, although the effect on potential benefits in data quality has not yet been systematically studied. However, for low-symmetry crystal systems, it is useful for our users to know that this is an option available to obtain higher data completeness which will only minimally affect the overall speed of SDU.

## 5. Summary

The implementation of fully automated data collection routines required substantial hardware and software upgrades to ensure dependable performance at the SLS MX beamlines. For example, extensive improvements of the TELL sample changer and TELL GUI have significantly increased our sample mounting reliability, while the installation of new top camera mounts (Fig. S1 of the supporting information) resulted in high ALC success rates. The modular architecture of TELL GUI and ALC enabled effective integration with SDU, which did not affect standard user operation in the initial stages of development.

The SDU software is best suited for (i) samples containing a single crystal mounted in an appropriately sized loop and (ii) well known and characterized crystallization systems such as crystals from high-throughput screening (HTS) or fragment-based drug discovery (FBDD) pipelines. The recent development of the SLS MX Fast Fragment and Compound Screening (FFCS) platform (Kaminski *et al.*, 2022 ▸) resulted in the in-house production of large quantities of fragment-soaked crystals for a number of academic and industrial targets, which were successfully measured with SDU. The systematic nature of SDU is also beneficial for beamline testing (*e.g.* TELL robot mounting and ALC) and methods development, such as the recent SmarGon MSC2 goniometer installation at the X06SA and X10SA beamlines.

**Figure 6:**
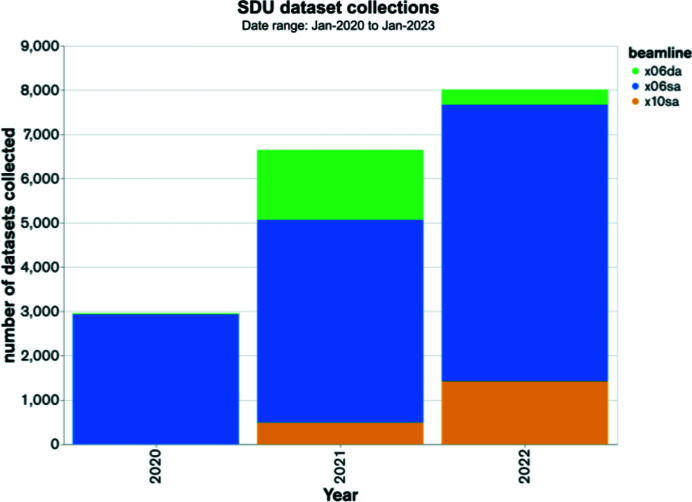
Number of datasets collected by SDU from 39 separate user groups since the first official release in 2020.

Our SDU development coincided with the requirement for fully remote operation imposed by the COVID-19 pandemic. The release of SDU was well received by our users, and the automated data collection mode is now utilized by several of our commercial partners. So far, SDU has reached an average throughput of 12 samples per hour at the X06DA bending magnet beamline, 20 samples per hour at the X10SA undulator beamline and 25 samples per hour at the X06SA undulator beamline (with recommended data collection parameters, Table S1). The X06SA and X06DA SDU software was officially released in the first half of 2021, and X10SA was released in the second half of 2021. The demand for SDU has been increasing since its initial release in 2020; from 17586 successful dataset collections across 39 separate user groups, 8006 of these were collected in 2022 (Fig. 6 ▸). We are actively working on expanding SDU capabilities; having recently implemented multi-orientation dataset collections, the next goal is investigating the integration of data collection for serial crystallography experiments. Future endeavors will explore the general trend towards smart automation, including the incorporation of machine learning to optimize capabilities and resources. There are ongoing efforts for using machine learning methods to optimize the accuracy of diffraction-based centering. This will be of great benefit as our system currently relies only on spot counts for identifying the best part of a crystal which is highly susceptible to errors when multiple crystal lattices are present (*e.g.* multiple crystals, cracks). Following the upcoming SLS upgrade we are aiming at providing a fully automated and self-aware beamline, capable of predicting component failure prior to its occurrence to ensure consistent high performance and reliability.

The programs described in this paper are available by written request to the corresponding authors.

## Supplementary Material

Figure S1. X06SA top camera mount schematics; Tables S1 to S3. DOI: 10.1107/S1600577523002631/yn5098sup1.pdf
