# Supplementary material for: SDU – software for high-throughput automated data collection at the Swiss Light Source
Source: J Synchrotron Radiat. 2023 Apr 12;30(Pt 3):538–45. doi: 10.1107/S1600577523002631 (PMC10161886; doi:10.1107/S1600577523002631)

JOURNAL OF SYNCHROTRON RADIATION

Volume 30 (2023)

Supporting information for article:

SDU: Software for high throughput automated data collection at the Swiss Light Source

Kate Mary Louise Smith, Ezequiel Panepucci, Jakub Wojciech Kaminski, Sylvain Aumonier, Chia-Yiang Huang, Deniz Eris, Dominik Buntschu, Nathalie Meier, Wayne Glettig, Katherine Evelyn McAuley, Meitian Wang, May Elizabeth Sharpe and Justyna Aleksandra Wojdyla

Table S1 Default data collection parameters for SDU collected data.

	**PXI/PXII**		**PXIII**	
**2D grid scan**	Exposure (sec)<br>Transmission (%)<br>Detector distance (mm)<br>Maximum grid size	0.02<br>100<br>300<br>4000	Exposure (sec)<br>Transmission (%)<br>Detector distance (mm)<br>Maximum grid size	0.1<br>100<br>300<br>4000
**Line scan**	Exposure (sec)<br>Transmission (%)<br>Detector distance (mm)<br>Maximum grid size	0.05<br>100<br>300<br>4000	Exposure<br>Transmission<br>Detector distance (mm)<br>Maximum grid size	0.1<br>100<br>300<br>4000
**Data collection**	Exposure (sec)<br>Oscillation (°)<br>Transmission (%)<br>Total range (°)<br>Detector distance (mm)	0.01<br>0.2<br>60<br>220<br>200	Exposure (sec)<br>Oscillation (°)<br>Transmission (%)<br>Total range (°)<br>Detector distance (mm)	0.1<br>0.2<br>100<br>220<br>200

Table S2 SDU diffraction threshold options available for determining the 'best position' on a crystal from Labelit analysis of grid scans.

**Diffraction**	**Total number of spots**	**Low resolution number of spots**	**Recommended use case**
strong	30	20	Well diffracting crystals
regular (default)	20	10	Standard measurements
weak	10	5	For weakly diffracting crystals (*e.g.* membrane proteins)

Table S3 Default data processing parameters from the hub for SDU collected data.

Parameter	Description	Hub Parameter	Default Value
Processing Pipeline	Defines method for final data processing in adp. Available options are: gopy, autoproc or xia2dials.	services_adp_method_key	gopy
Resolution Cutoff Value	Value used for resolution cutoff in the final processing.	adp_params_rescut_cc	30
		adp_params_rescut_is	1.0
Resolution Cutoff Key	Defines which resolution cutoff is used in the final step of adp. Available options are: is or cchalf	adp_params_rescut_key	is
Trusted High	Defines a trusted region of detector for xds processing.	adp_params_trustedhigh	1.21
User Resolution	Used in adp for the final step of processing in gopy.	adp_params_userresolution	None
No Anomalous	Defines whether Friedel pairs are False or True during xds processing. Used by adp for gopy or autoPROC.	adp_params_noAno	false
Reference Data	Defines path to reference dataset for adp.	adp_params_referencedata	None
Space Group Number	Space group used by adp when processing with gopy, autoPROC or xia2dials.	adp_params_sgnumber	None
Cell parameters	Cell parameters used by adp when processing with gopy, autoPROC or xia2dials.	adp_params_cellparams	{ a : 77.7, b : 77.7, c : 77.7, alpha : 90, beta : 90, gamma : 90}
Autoproc Full	Allows processing with the full version of autoPROC without use of fast.macro	adp_params_autoprocFull	false
FFCS Campaign	Enabled creation of linked mtz files in the FFCS campaign folder.	adp_params_ffcs_campaign	false
PDB Model	PDB model used for automated dimple run.	adp_params_pdbmodel	None

Figure S1 X06SA top camera mount schematics.

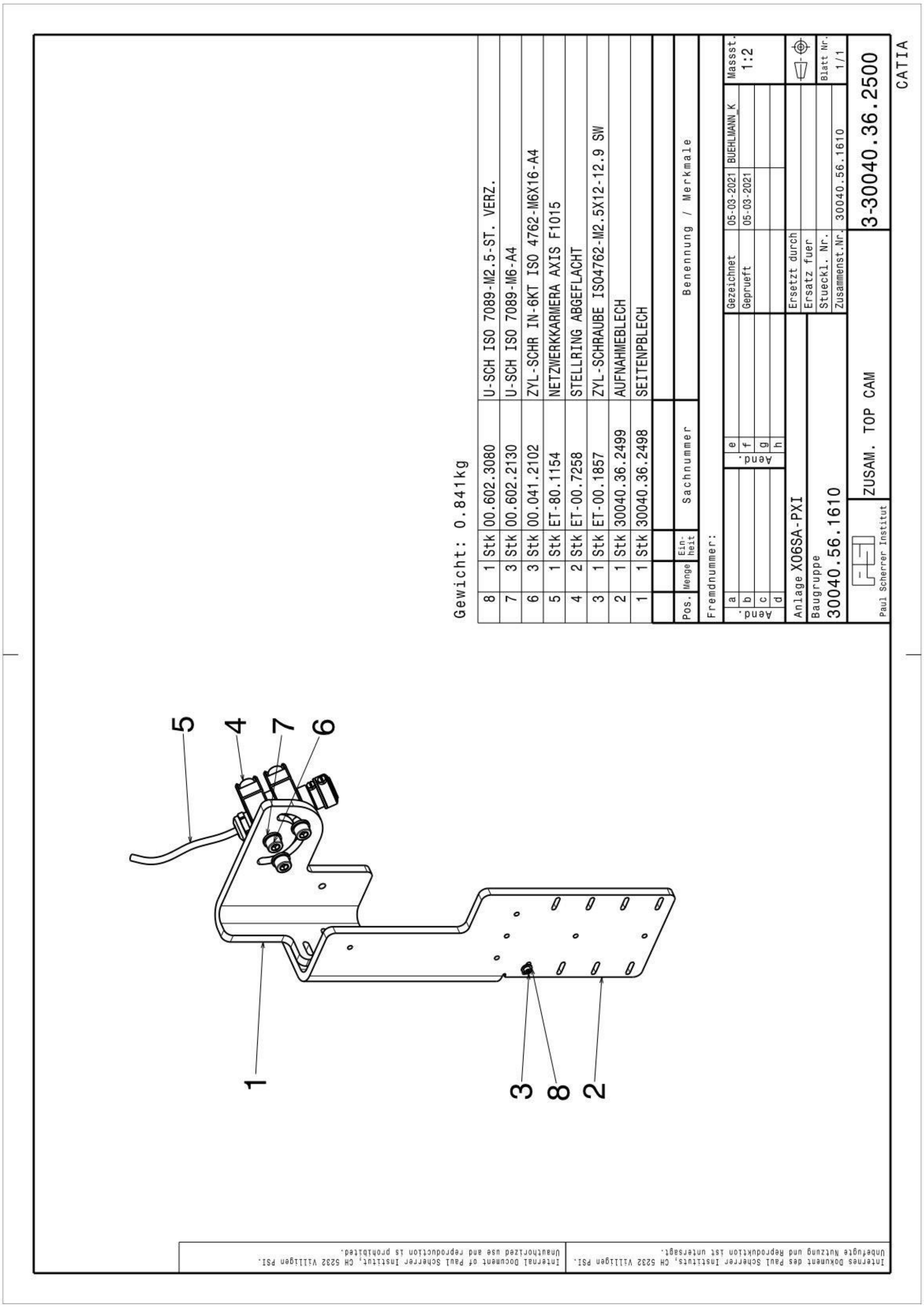

Supplement: Supplementary file 1 [file s-30-00538-sup1.pdf]
